# Supplementary material for: Controllable conversion of quasi-freestanding polymer chains to graphene nanoribbons
Source: Nat Commun. 2017 Mar 13;8:14815. doi: 10.1038/ncomms14815 (PMC5355836; doi:10.1038/ncomms14815)
Supplement: Supplementary Information — Supplementary Figures, Supplementary Note and Supplementary References. [file ncomms14815-s1.pdf]

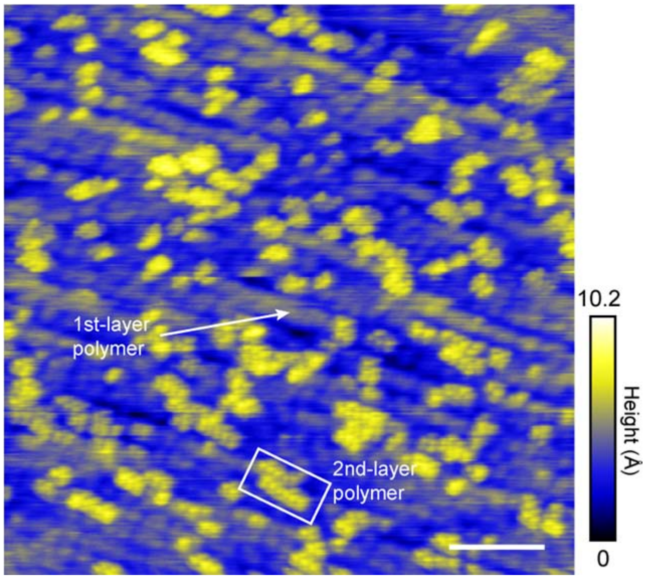

Supplementary Figure 1 | Formation of the polymer chains after 470 K annealing both on the 1st and 2nd layers with a coverage θ > 1 (setpoint: sample voltage V_s = −2 V, tunnelling current I_t = 100 pA). Scale bar, 4 nm.

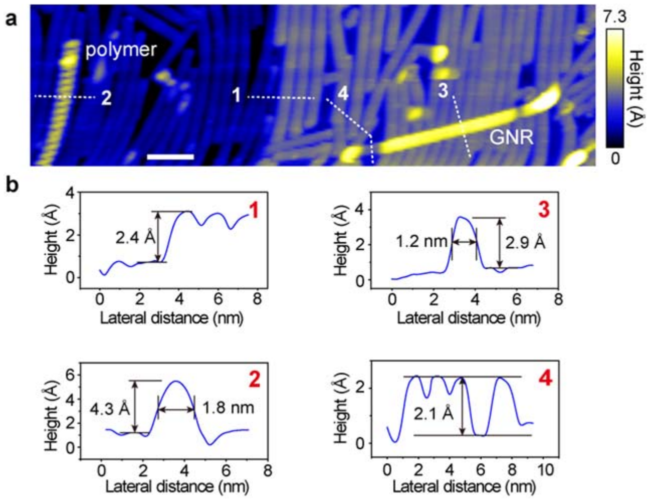

Supplementary Figure 2 | Structure characterization of the 1st- and 2nd-layer armchair graphene nanoribbons with a width of seven carbon (7-aGNRs), and the 2nd-layer polymer chains. **a**, Scanning tunnelling microscopy (STM) image showing a 2nd-layer polymer and GNR ($V_s = -2$ V, $I_t = 100$ pA) after 670 K annealing, same as Fig. 2a. Scale bar, 5 nm. **b**, Profiles 1—4 along the dashed lines in (a). Profile 1: Across 1st-layer GNRs on two different terraces of Au(111), giving a terrace height of 2.4 Å of Au(111). Profile 2: Across a 2nd-layer polymer chain, giving the height and width (full width at half maximum) of 4.3 Å and 1.8 nm, respectively. Profile 3: Across a 2nd-layer GNR, giving the height and width of 2.9 Å and 1.2 nm, respectively. Profile 4: Across the 1st-layer GNRs and the part in contact with Au substrate of the 2nd-layer GNR, which show the similar height.

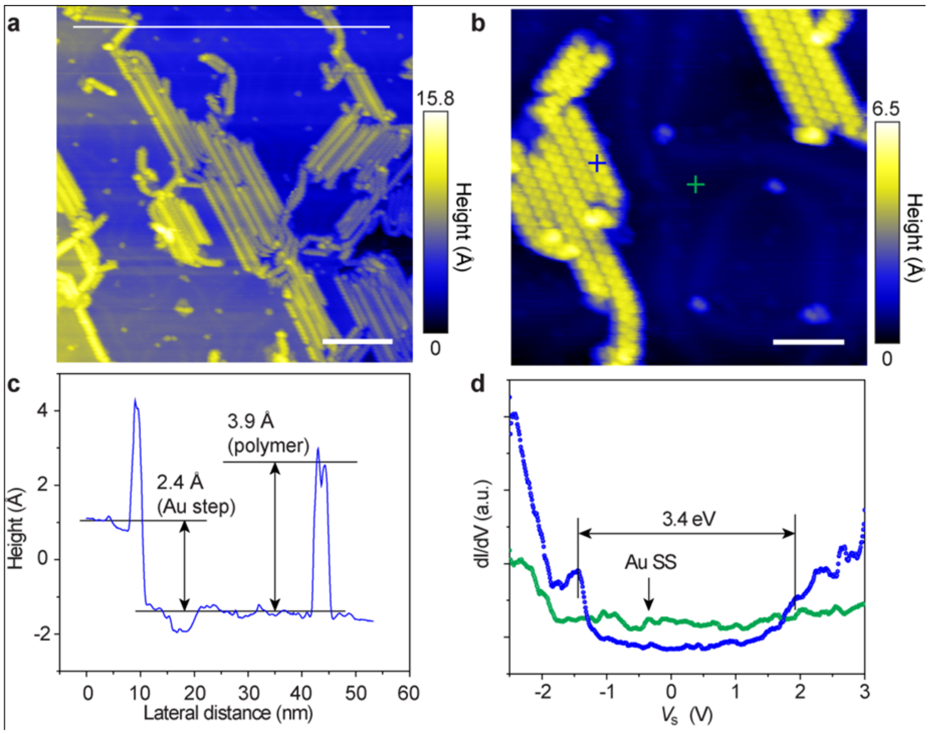

Supplementary Figure 3 | Height and Electronic properties of the 1st-layer polymer. **a**, STM image showing the polymer chains on Au with a coverage $\theta \sim 0.4$, obtained after 470 K annealing. Scale bar, 12 nm. **b**, High-resolution STM image of the 1st-layer polymer chains. Scale bar, 4 nm. **c**, Profile along the white line in (**a**), showing the height of the 1st-layer polymer chains of about 3.9 Å. **d**, Differential conductance, d*I*/d*V*, curves acquired at the cross marked sites in (**b**) (without shift). The blue curve acquired on the 1st-layer polymer (blue cross), showing a bandgap of about 3.4 eV. The green curve acquired on Au(111) substrate (green cross), showing the typical Au surface states (SS) (marked with an arrow). Setpoint in (**a**), (**b**) and (**d**): $V_s = -2$ V, $I_t = 100$ pA.

For a deposition coverage $\theta < 1$ of the 10,10'-dibromo-9,9'-bianthryl (DBBA) molecules, the polymer chains prefer to form islands after 470 K annealing. Supplementary Figs. 3a and 3b show the polymer islands with a coverage $\theta \sim 0.4$. In Supplementary Fig. 3c, the 1st-layer polymer displays a height of about 3.9 Å, which is slightly smaller than that of the 2nd-layer polymer (~4.3 Å). Supplementary Fig. 3d shows the d*I*/d*V* curves acquired both on the polymer and Au substrate. The one on the polymer shows a bandgap of about 3.4 eV, while the one on the Au exhibits clear features of Au surface states (SS). The smaller bandgaps for the 1st-layer polymer and the 7-aGNR (Fig. 2) compared to their 2nd layers, indicate strong substrate interactions due to direct adsorption on Au substrate[1].

Due to a higher work function of Au(111) (~5.2 eV) than 7-aGNR (~4.7 eV)[1], electrons from polymer (with an effective work function ~4.0 eV) are energetically easier to transfer to Au than to GNR. This is consistent with our experimental observations that the cyclodehydrogenation happens more easily while the polymer is adsorbed on Au as compared to the 7-aGNR during thermal annealing. The smaller difference of work function between the polymer and GNR should be the reason why the 2nd-layer polymer can be converted to GNR at the end. In a previous effort of growing GNR with DBBA molecules on an insulating TiO_2(011)-(2×1) surface[2], only polymerization was achieved but without cyclodehydrogenation, which might be attributed to the low work function of TiO_2 (~4.2 eV[3] or lower when adsorbates are involved[4]).

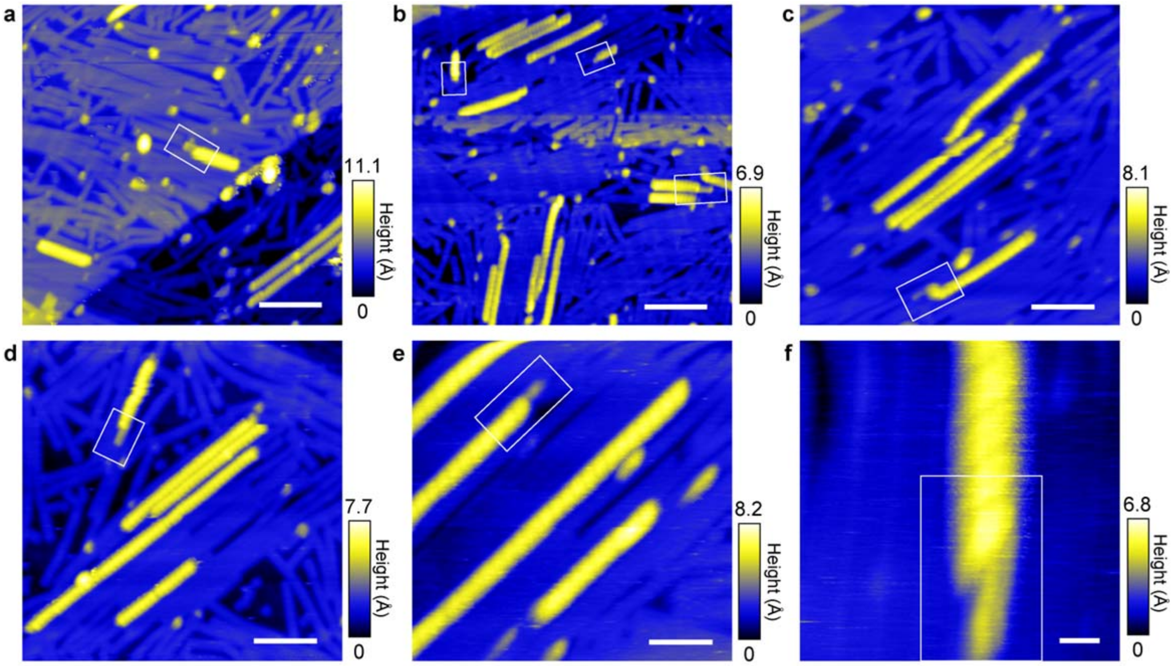

Supplementary Figure 4 | More STM images showing the 2nd-layer polymers with GNR tails, marked with white boxes after 670 K annealing. Setpoint: (**a**) $V_s = -2$ V, $I_t = 50$ pA, (**b**) $V_s = -2$ V, $I_t = 60$ pA, (**c**) $V_s = -2$ V, $I_t = 50$ pA, (**d**) $V_s = -2$ V, $I_t = 10$ pA, (**e**) $V_s = -2$ V, $I_t = 30$ pA, (**f**) $V_s = -2$ V, $I_t = 100$ pA. Scale bars, (**a**) 13 nm, (**b**) 12 nm, (**c**) 10 nm, (**d**) 10 nm, (**e**) 7 nm, (**f**) 1 nm.

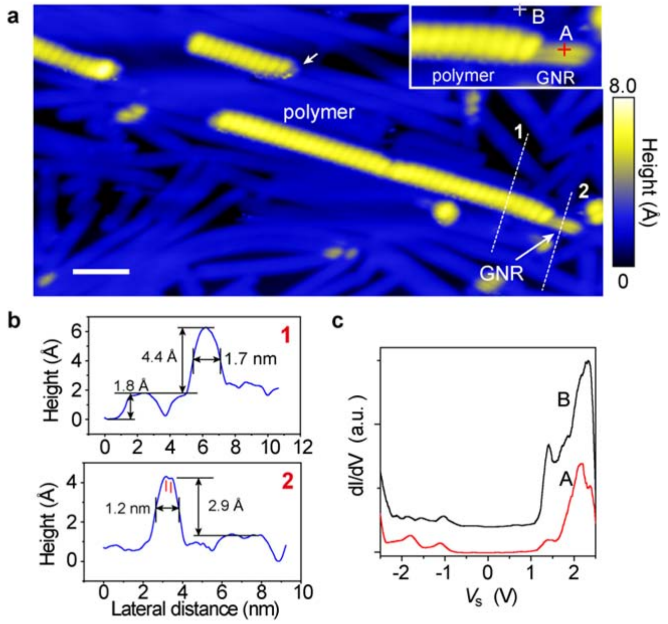

Supplementary Figure 5 | Structure and electronic properties characterization of a 2nd-layer polymer chain with a GNR tail after 670 K annealing. **a**, STM image showing formation of intraribbon heterojunctions of the polymer chains with GNR tails, marked with white arrows (V_s = −2 V, I_t = 100 pA). Inset: magnified image of the polymer with a GNR tail region. Scale bar, 5 nm. **b**, Profiles 1—2 of the polymer chain and the GNR tail along the dashed lines in (**a**). The 2nd-layer GNR tail displays a height of about 2.9 Å, while the 1st-layer GNR is about 1.8 Å and the 2nd-layer polymer about 4.4 Å. In Profile 2, red lines mark the features of the edge states. **c**, dI/dV curves acquired correspondingly on the cross marked sites (A and B) in the inset, respectively (V_s = −2 V, I_t = 100 pA). The 2nd-layer GNR tail has very similar electronic properties to the 1st-layer one, but a slightly larger bandgap.

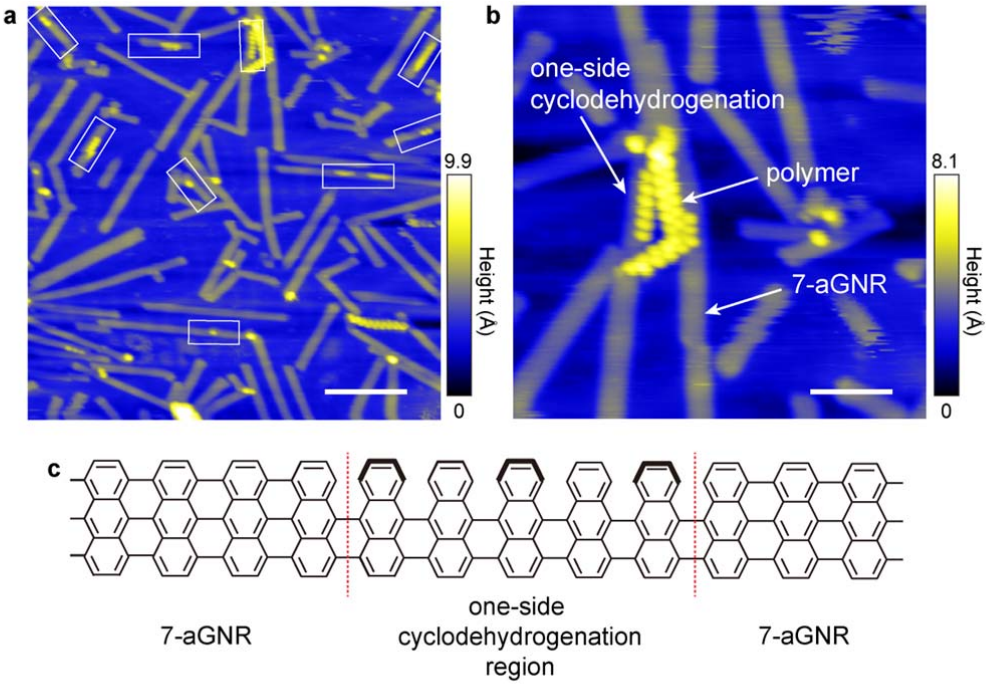

Supplementary Figure 6 | One-side-domino cyclodehydrogenation for the 1st-layer polymers. a, Large-area STM image showing the sample after 600 K annealing for 20 min. White boxes mark the one-side converted parts in 7-aGNRs. Scale bar, 10 nm. **b**, Zoom-in STM image showing the 7-aGNRs, one polymer chain, and one GNR after partial one-side cyclodehydrogenation. Scale bar, 4 nm. Setpoint in (**a**) and (**b**): V_s = −2 V, I_t = 100 pA. **c**, Schematic of the formed intraribbon heterojunction after one-side cyclodehydrogenation. The one-side-domino cyclodehydrogenation is significantly different from the domino-like conversion of the 2nd-layer polymer.

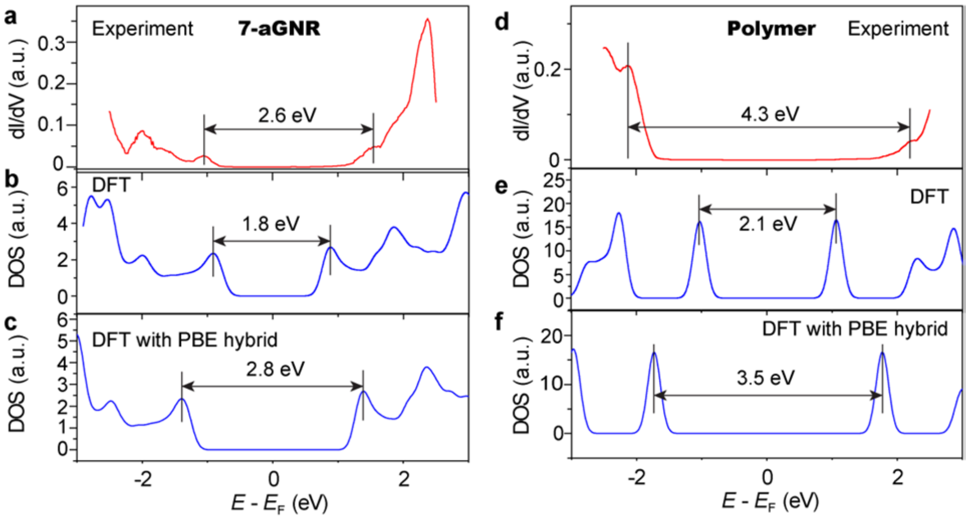

Supplementary Figure 7 | Comparison of bandgaps between experiment and theory. a, Experimentally acquired d*I*/d*V* spectrum of the 7-aGNR. **b**, Density functional theory (DFT) calculated density of states (DOS) of the 7-aGNR. **c**, DFT with Perdew-Burke-Ernzerhof (PBE) hybrid exchange correlation calculated DOS of the 7-aGNR. **d**, Experimentally acquired d*I*/d*V* spectrum of the polymer. **e**, DFT calculated DOS of the polymer. **f**, DFT with PBE hybrid exchange correlation calculated DOS of the polymer. During the calculations, the GNR and polymer are in vacuum.

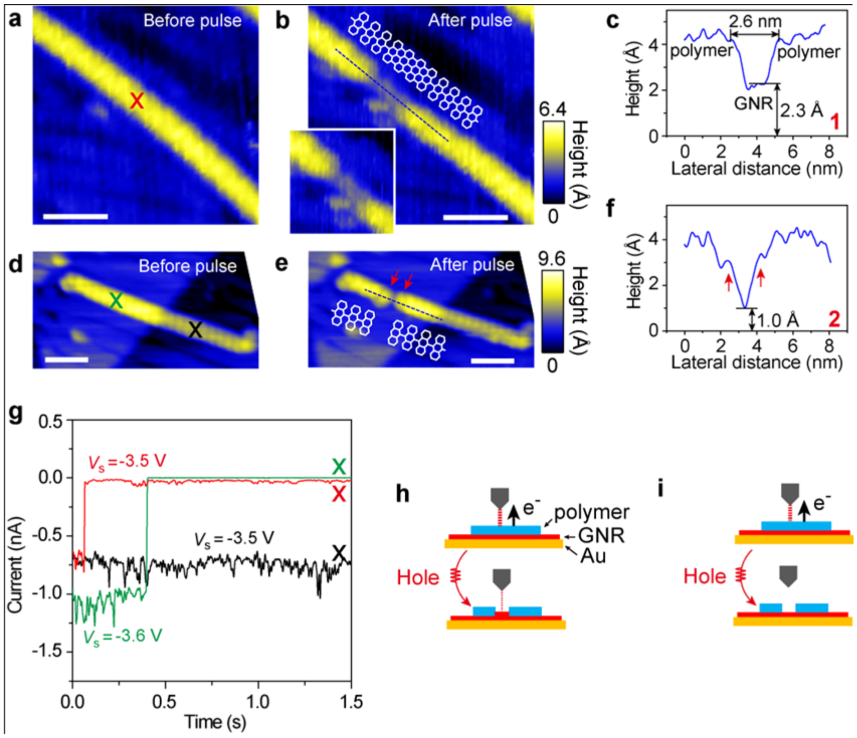

Supplementary Figure 8 | Hole-involved cyclodehydrogenation and breaking of the polymer chains induced by an STM tip with feedback loop off. a, STM images (V_s = −2 V, I_t = 100 pA) of a 2nd-layer polymer chain before and, **b**, after the pulse treatment with V_s = −3.5 V for time t = 1.5 s to form an GNR segment in the polymer with the feedback loop off, while the tip is held at setpoint V_s = −2 V and I_t = 100 pA. Inset: Magnified image of the formed segment with narrower apparent width and lower height, where the typical intensity-enhanced states at the GNR edges are found, similarly to Fig. 2a. **c,** Profile along the dashed line in (**b**), normalized to the polymer height, which gives the height of about 2.3 Å for the newly formed segment. These results indicate the formation of GNR in the polymer chain. The length of the converted segment is about 2.6 nm, which is about triple the period of the polymer chain. Thus, we can propose that the pulse has converted two adjacent periodical units of the polymer to GNR, as shown in the inset of (**b**). **d**, STM images of a 2nd-layer polymer chain before and, **e**, after the pulse treatment (V_s = −3.6 V, t = 1.5 s) that broke the polymer, while the tip is held at setpoint V_s = −2 V and I_t = 100 pA with feedback loop off (V_s = −2 V, I_t = 100 pA). The red arrows mark the newly formed ends. **f**, Profile along the dashed line in (**e**), normalized to the polymer height. The disconnected polymer segments display a much lower height of about 1.0 Å. Red arrows in (**f**) mark the features corresponding to the newly formed ends in (**e**), which are slightly lower than the bulk part of the polymer chain, indicating of the formation of unsaturated C radicals. **g**, I_t-t curves acquired from

the cross marked sites in (**a)** and (**d)**. **h**, Schematic of the tip-induced conversion of polymer to GNR, and **i**, cutting of polymer chains by hole injection. When the polymer is converted to GNR, the current will drop to a small value (red), because of the increased tip-sample distance, as illustrated in Supplementary Fig. 4h. In contrast, the current will drop to zero (green) while cutting the polymer, because the tip-sample distance is beyond the tunnelling distance, as illustrated in Supplementary Fig. 4i. All scale bars, 4 nm.

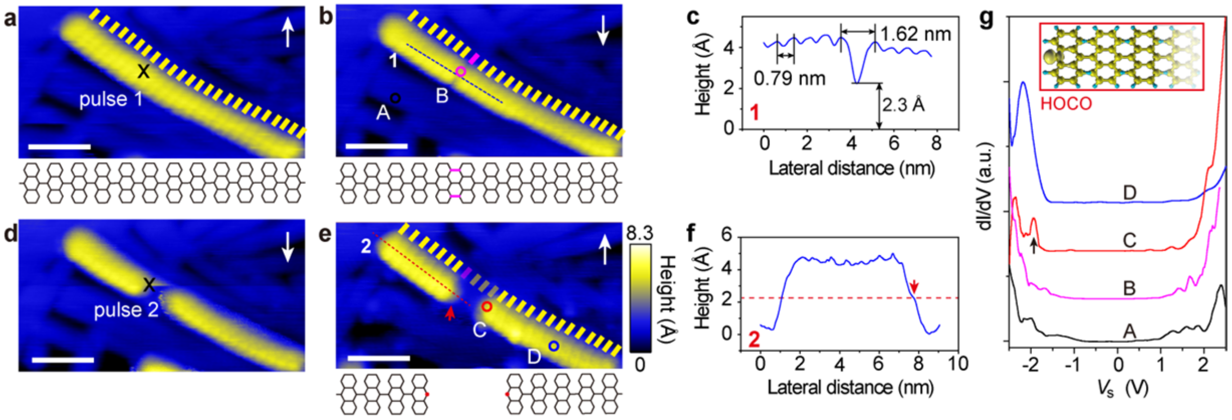

Supplementary Figure 9 | Tip-induced manipulation of the polymer chains by pulses of holes with feedback loop on. a, STM image of a polymer chain before and, **b** after applying a pulse (pulse 1: $V_s = -2.5$ V, $I_t = 50$ pA, $t = 10$ ms) at the cross marked site (respective schematic structure models are also given). **c**, Profile along the dashed line in (**b**), showing a width of about 1.62 nm of the defect induced by pulse 1, which is about twice the period in the polymer. **d**, A second pulse (pulse 2: $V_s = -2.5$ V, $I_t = 50$ pA, $t = 10$ ms) was applied to the cross marked site during the downward scanning. **e**, A sequence image after the pulse. Setpoint in (**a**)-(**b**) and (**d**)-(**e**): $V_s = -2$ V, $I_t = 50$ pA. The short lines in (**a**), (**b**) and (**e**) mark the periodic units in the polymer chain, pink for GNR and transparent for disappeared. White arrows in (**a**)-(**b**) and (**d**)-(**e**) represent the scanning directions. **f**, Profile along the red line in (e), red arrow marks the shoulder feature. **g**, dI/dV curves acquired at the circle marked sites in (**b**) and (**e**), with an arrow marked the peak emerging from the cut end. Inset: Charge density distribution (acquired as the square of wavefunction) of the highest occupied crystal orbital (HOCO) of the polymer with an unsaturated C atom at the end, showing localized states at the C radical. All scale bars, 4 nm.

Tip treatment with the STM feedback loop on can also convert the polymer to GNR. After applying a pulse of $V_s = -2.5$ V and $I_t = 50$ pA for $t = 10$ ms at the cross marked site in Supplementary Fig. 9a, a defect structure is formed in Supplementary Fig. 9b. In Supplementary Fig. 9c, the defect width is about 1.62 nm, nearly twice the period of the polymer. Similarly to Fig. 4, the structure can be assigned to the formation of a GNR segment, as illustrated in the lower panel of Supplementary Fig. 9b. Another pulse with the same parameters is applied to the GNR segment (Supplementary Fig. 9d) during the downward scanning. The pulse blows away the GNR segment together with another three polymer units, as illustrated in Supplementary Fig. 9e. At the newly formed end of the polymer segments, a shoulder feature is found (red arrows). The feature

can be clearly seen from the profile in Supplementary Fig. 9f, and is obviously absent at the normal polymer ends. This structure is consistent with a C radical, as illustrated in the lower panel of Supplementary Fig. 9e. The dI/dV curves in Supplementary Fig. 9g can further confirm the assignments. The one acquired at the GNR segment is similar to that on 1st-layer GNR. The curve from the cut end shows a distinct peak (arrow) compared to that in the polymer chain, indicating the localized states caused by the C radical. These features can be well reproduced by the simulated charge density distribution of the HOCO of the polymer with an unsaturated C atom at the end, where clearly localized states can be found at the C radical.

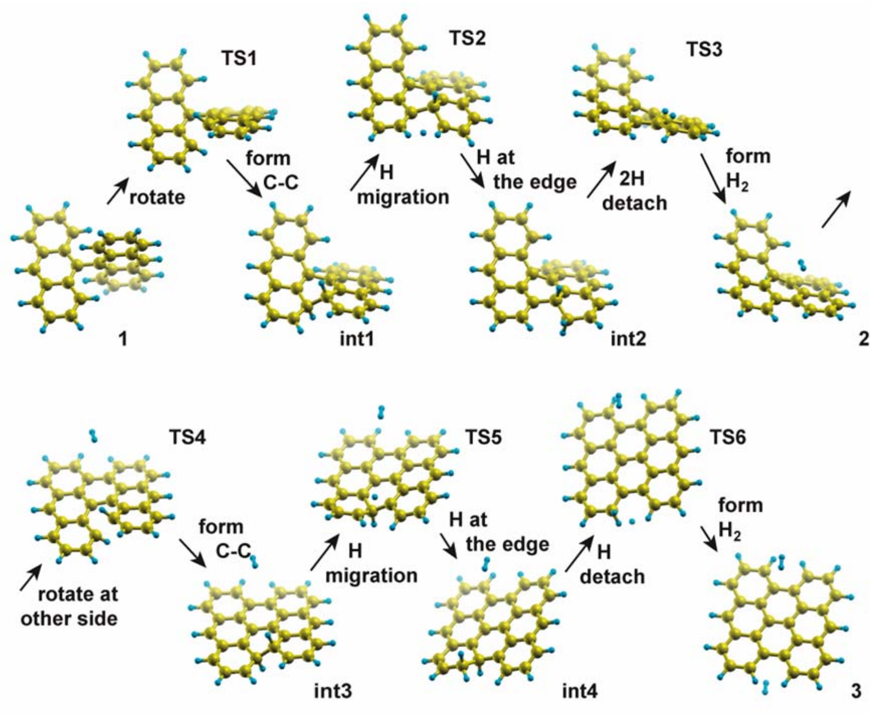

Supplementary Figure 10 | Detailed reaction path for the cyclodehydrogenation of a bianthrylene molecule. 1 refers to the initial state, 2 refers to the state after one-side cyclodehydrogenation, and 3 refers to the final state, while int1–int4 are the intermediates, and TS1–TS6 are the transition states. Note, in order to show the reaction path on the other side (State TS4 to State 3), the perspective is different from that from State 1 to State 2.

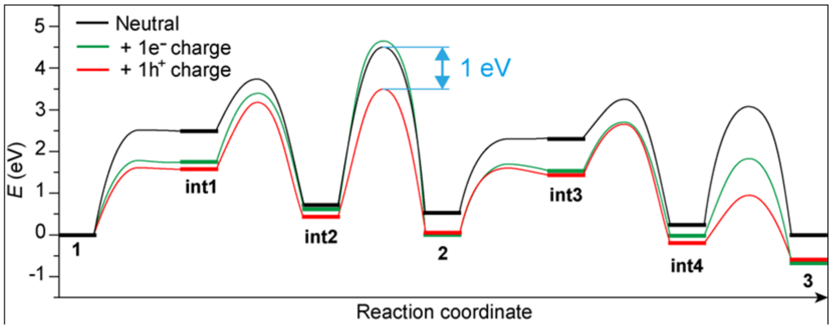

Supplementary Figure 11 | Energy diagrams of cyclodehydrogenation in vacuum with single-charge injection. The energy diagrams for neutral (black), single-electron (green) and single-hole (red) assisted bianthrylenes, respectively, are calculated with the same reaction path in the main text (Fig. 4d). With single-hole injection, the energy barrier can be reduced by about 1.0 eV from 4.5 eV (neutral) to 3.5 eV, while with single-electron injection, the barrier does not decrease.

We find that the barrier is reduced from 4.5 eV for the neutral case to 3.5 eV with a single-hole injection, where the reduction of 1.0 eV is much smaller than that of two-hole system (1.7 eV, Fig. 4d). Moreover, there is a negligible energy barrier to prevent the int1 state from going back to state 1, unlike the two-hole injection case shown in Fig. 4d. In addition, the much reduced barrier for the two-hole injection system (2.8 eV) is comparable to the STM biases used to trigger the polymer-to-GNR reaction (–2.5 eV to –2.8 eV). Thus, we believe the two-hole injection mechanism is more reasonable here. For the Scholl reaction mechanism, a second hole is needed to rearomatize the system too[5].

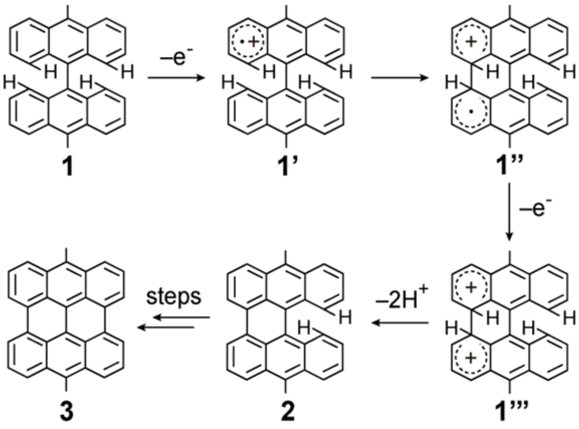

Supplementary Figure 12 | Reaction path for the hole-assisted cyclodehydrogenation in one polymer unit based on a Scholl reaction in a solution phase. The hydrogen atoms involved in this path are highlighted. The elimination of H in the form of either H_2 or $2H^+$ is dependent on the reaction environment, the gas phase in the ultrahigh vacuum (UHV) of the present study or the solution phase in the Scholl reaction[5]. In the gas phase, our calculations for the bianthrylene case with q=+2 indicate that the elimination of H_2 is energetically more favorable than the elimination of $2H^+$. This is because charges prefer to be delocalized onto a greater domain of bianthrylene instead of being localized on two H^+. However, for the Scholl reaction in a solution phase, the localized charges on H^+ can be significantly stabilized by solvation energy. Thus we show H_2 elimination in Fig. 4c, and $2H^+$ elimination in Supplementary Fig. 12.

Supplementary Note 1

Yield and optional parameters of tip treatments for inducing polymer-to-GNR reaction. During the experiment, we find that pulses with negative bias (hole injection) can both trigger polymer-to-GNR reaction and damage the polymer chain, while those with positive bias (electron injection) can just damage the polymer chain. In about 500 experimental treatments with negative bias, 20% of them can change polymer chains, with a ratio of 1:1 between damaging the polymer and triggering the cyclodehydrogenation reaction. The most efficient parameter of pulses for the polymer-to-GNR reaction is sample bias V_s in the range of −2.5 to −2.8 V, tunnelling current I_t in the range of 50 to 100 pA at the setpoint of $V_s = -2$ V and $I_t = 100$ pA, with a cyclodehydrogenation reaction of about 50% in total.

Based on our nudged elastic band (NEB) calculations[6], a much higher energy barrier exists for the C–C bond formation with electron injections than hole injections. To overcome the higher energy barrier for electrons, a higher bias is needed, which increases the probability of damaging the polymer. Indeed the experimental results show that the polymer is damaged by high positive bias pulses, but not changed by low positive bias pulses.

Supplementary References:

1. Liang, L. & Meunier V. Electronic structure of assembled graphene nanoribbons: Substrate and many-body effects. *Phys. Rev. B* **86**, 195404 (2012).
2. Kolmer, M. *et al.* Polymerization of Polyanthrylene on a Titanium Dioxide (011)-(2×1) Surface. *Angew. Chem.* **125**, 10490-10493 (2013).
3. Imanishi, A., Tsuji E. & Nakato Y. Dependence of the Work Function of TiO_2 (Rutile) on Crystal Faces, Studied by a Scanning Auger Microprobe. *J. Phys. Chem. C* **111**, 2128-2132 (2007).
4. Onda, K., Li B. & Petek H. Two-photon photoemission spectroscopy of TiO_2 (110) surfaces modified by defects and O_2 or H_2O adsorbates. *Phys. Rev. B* **70**, 045415 (2004).
5. Grzybowski, M., Skonieczny K., Butenschön H. & Gryko D. T. Comparison of Oxidative Aromatic Coupling and the Scholl Reaction. *Angew. Chem. Int. Ed.* **52**, 9900-9930 (2013).
6. Henkelman, G., Uberuaga B. P. & Jónsson H. A climbing image nudged elastic band method for finding saddle points and minimum energy paths. *J. Chem. Phys.* **113**, 9901-9904 (2000).
